# Supplementary material for: Evaluation of Atypical Chemokine Receptor Expression in T Cell Subsets
Source: Cells. 2022 Dec 16;11(24):4099. doi: 10.3390/cells11244099 (PMC9776531; doi:10.3390/cells11244099)
Supplement: Supplementary file 1 [file cells-11-04099-s001.zip › cells-2074448-supplementary.pdf]

Supplemental Material

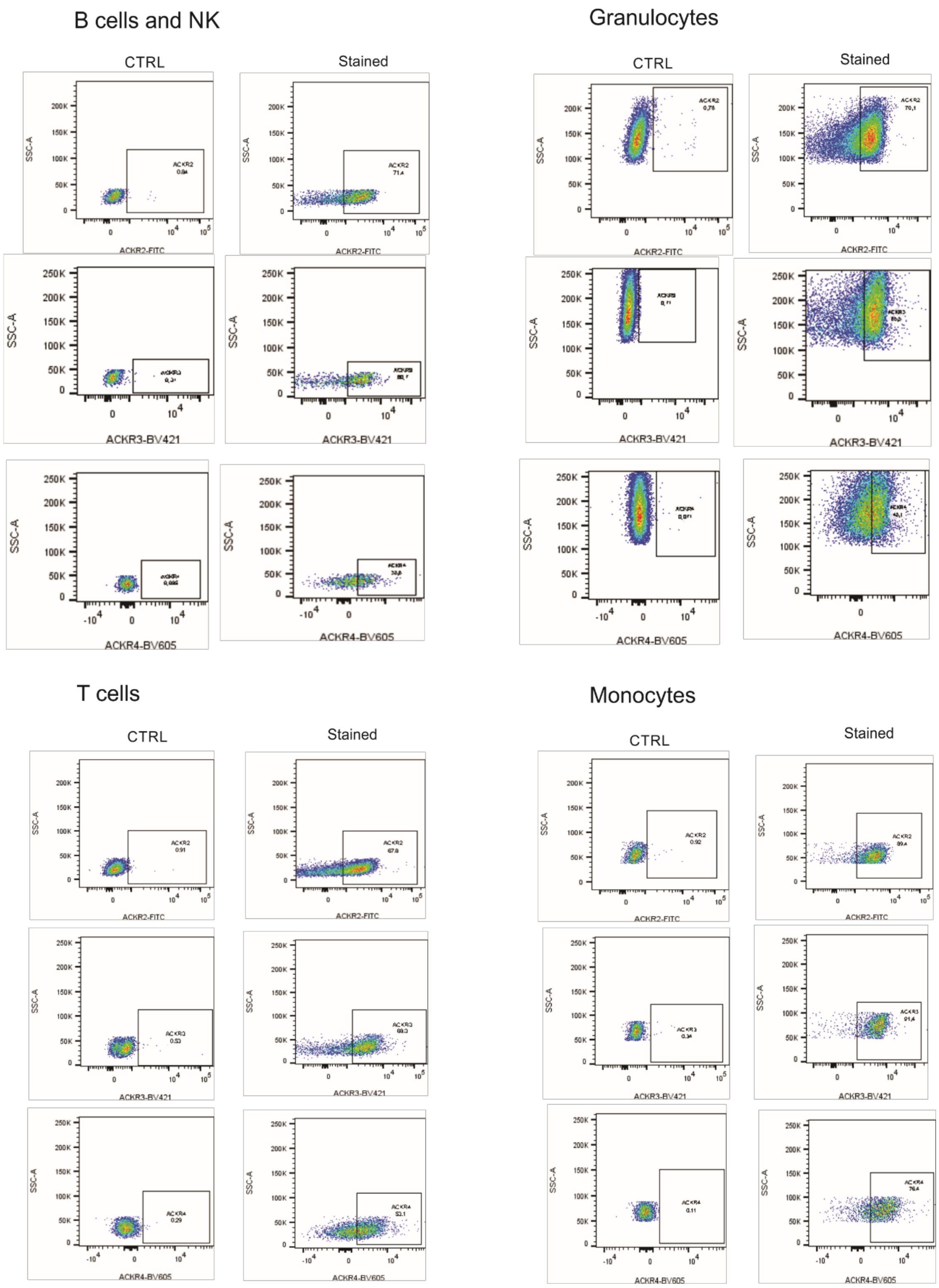

Figure S1 – Gating strategy for atypical receptors (ACKR2, ACKR3 and ACKR4) on circulating leukocytes . Flow cytometry dot plots displaying each population as B cells/NK cells, Granulocytes, T cells and Monocytes on negative control and the stained populations for ACKR2, ACKR3 and ACKR4.

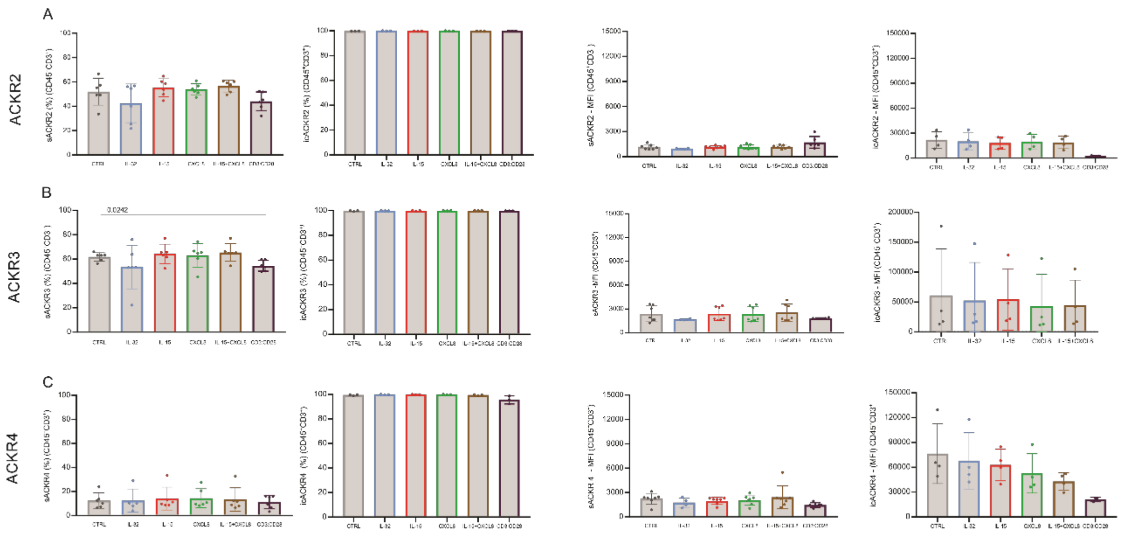

Figure S2 – Expression of ACKRs (ACKR2, ACKR3 and ACKR4) on surface and in intracellular compartment of T cells. (A) Graphs with percentage of T cells expressing ACKR2 on the surface, graph with T cells expressing ACKR2 in the intracellular compartment, graph with the MFI of ACKR2 for the surface staining and the graph with the MFI for intracellular staining. (B) Graphs with percentage of T cells expressing ACKR3 on the surface, graph with T cells expressing ACKR3 in the intracellular compartment, graph with the MFI of ACKR3 for the surface staining and the graph with the MFI for intracellular staining. (C) Graphs with percentage of T cells expressing ACKR4 on the surface, graph with T cells expressing ACKR4 in the intracellular compartment, graph with the MFI of ACKR4 for the surface staining and the graph with the MFI for intracellular staining.
